# In Ovo Feeding of Arginine, Leucine, and Methionine in Broiler Breeders’ Eggs During Summer: Effects on Hatchability and Chick Oxidation, Inflammation, and Apoptosis

**DOI:** 10.3390/ani15131930

**Published:** 2025-06-30

**Authors:** Huan Ge, Zhenwu Huang, Jinghai Feng, Minhong Zhang

**Affiliations:** State Key Laboratory of Animal Nutrition, Institute of Animal Sciences, Chinese Academy of Agricultural Sciences, Beijing 100193, China; 82101225435@caas.cn (H.G.); 2019105026@njau.edu.cn (Z.H.); fjh6289@126.com (J.F.)

**Keywords:** summer, late laying period, broiler chicken, in ovo feeding, methionine, arginine, leucine, oxidation–inflammation–apoptosis, growth and development

## Abstract

Summer high temperatures (27–38 °C, more than 7 days) readily induce heat stress in late-laying broiler breeders, which impedes offspring growth and development. This study aimed to provide a scientific basis for improving the offspring development of late-laying broiler breeders during summer. Six hundred fertilized eggs from 50-week-old LiFeng broiler breeders were divided into five treatment groups (non-injected, NaCl-injected, 6 mg/egg L-arginine-injected, 3.5 mg/egg L-methionine-injected, 8.4 mg/egg L-leucine-injected), with six replicates per group and 20 eggs per replicate. Embryos were incubated for 21 days and chicks raised for 21 days post-hatch. Methionine injection significantly enhanced hatchability (+5.8%), increased daily chick weight gain (+8.8%), reduced serum urea nitrogen (−53.13%), decreased inflammatory cytokine levels, elevated antioxidant enzyme activities, lowered malondialdehyde content (−47.99%), and suppressed expression of inflammatory and apoptotic pathway genes. The comprehensive effect of methionine was the best among the three amino acids when injected into chicken embryos. Methionine promoted protein synthesis, enhanced antioxidant and anti-inflammatory capacities, and consequently improved chick growth performance.

## Simple Summary

High summer temperatures (27–38 °C, more than 7 days) readily induce heat stress in late-laying broiler breeders, leading to reduced egg hatchability, impaired embryonic development, and compromised chick growth performance. Currently, no targeted nutritional interventions for summer-laid eggs from late-laying broiler breeders have been reported. This study systematically investigated the effects of embryonic injection with three amino acids (arginine, leucine, and methionine) on egg incubation and chick growth to establish a theoretical basis for improving offspring development in summer-laid eggs. The results demonstrated that methionine injection significantly enhanced hatchability and promoted chick growth, while arginine and leucine exhibited no significant effects.

## 1. Introduction

The nutritional level within the egg determines the development of broiler chicken [1]. In the later incubation period, chicken embryos experience intense physiological alterations and consume a considerable amount of energy, resulting in a deficiency in glycogen and protein, along with a decrease in amino acid content, which fails to satisfy the embryo’s demands [2]. Insufficient nutrition during the late stage of incubation might constrain the development of the chicken embryo and the post-hatching growth performance of the chicks [1]. Meanwhile, in the context of poultry breeding, chicks are usually not provided with feed until 24 to 48 h after hatching [3]. Such a practice leads to malnutrition among newly hatched chicks and an elevated mortality rate [4]. In ovo feeding (IOF) is an effective method to improve egg nutrients and to address early development difficulties [5,6,7]. After more than 40 years of research and development, IOF has become a mature alternative early nutritional control measure [8]. A growing number of studies indicate that supplementing nutrients to chicken embryos during the late incubation stage can ameliorate the nutritional status between the late incubation period and the first feeding [6,9].

Arginine (Arg), methionine (Met), and leucine (Leu) are critical amino acids with distinct roles in poultry development. Arg regulates nutrient metabolism by acting as a glucogenic precursor [10,11], modulating energy metabolism, and stimulating hormonal activity [10]. Experimental evidence demonstrates that in ovo Arg administration enhances duodenal Villus Height (VH) while reducing Crypt Depth (CD) in broiler embryos and post-hatch chicks [6], and improves hepatic reserves in turkey embryos to support post-hatch growth [10]. Similarly, Met emerges as the primary limiting amino acid in poultry diets [12], fulfilling vital functions including glutathione synthesis, DNA methylation regulation [13,14], tissue protein formation [14], and suppression of neonatal gluconeogenesis [15]. Embryonic Met supplementation promotes intestinal morphogenesis through increased villus dimensions, goblet cell density, and enhanced embryonic growth [16,17]. The branched-chain amino acid Leu contributes to developmental regulation by accelerating hatching timelines [18], while demonstrating metabolic modulation capabilities through amino acid/lipid metabolism optimization and thermotolerance enhancement in chickens [19,20,21,22,23,24].

Hens exposed to high temperatures may undergo extreme physiological disorders, such as reduced feed efficiency, decreased egg-laying rate, and lowered egg quality, such as egg brightness, yolk weight and percentage, and low specific gravity [25,26]. Meanwhile, as the ovaries age and the quality of oocytes deteriorates, laying hens in the late laying period commonly experience phenomena such as reduced egg-laying rate and declined egg quality [27,28,29,30,31]. Given the current situation of large-scale production of fertilized eggs under high-temperature adversity, it is urgent to explore a suitable nutritional intervention method. However, no studies have yet reported the effects of amino acids on fertilized eggs produced by broiler breeders during the late laying phase under high-temperature seasonal conditions. Therefore, in this experiment, IOF was performed on the eggs of broiler breeders in the late laying period during summer high temperatures, with Arg, Met and Leu injected respectively, to explore the most appropriate amino acid supplementation method for such eggs.

## 2. Materials and Methods

### 2.1. Hatching and Incubation

**Figure 1:**
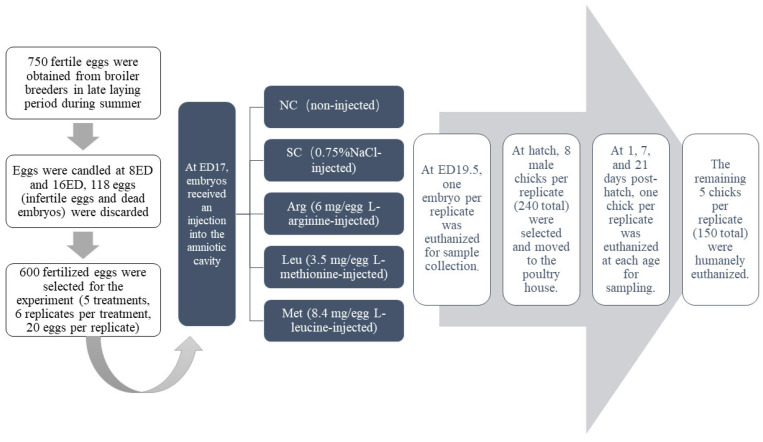
Flow chart of the experimental design process. ED: days of embryonic age.

The experimental design process is shown in Figure 1. A total of 750 fertile eggs (average weight: 64.25 g) were collected from 50-week-old LiFeng broiler breeders at a breeding farm in Henan Province, China. The breeder house was maintained at 28–32 °C with 92–97% relative humidity. Eggs were incubated in automatic incubators (2112 type incubator, Limin Comp., Dezhou, China) following the manufacturer’s protocol. Unfertilized and malformed eggs (*n* = 118) were removed after candling on Days 8 and 16 of incubation. On Day 16 of incubation, 600 viable eggs were randomly allocated to 5 treatment groups with 6 replicates per group (20 eggs per replicate). The 5 treatment groups included the non-punctured control group (NC group); 0.75% saline-injected control group (SC group); 7.0 g/L L-methionine solution-injected group (Met group); 16.8 g/L L-leucine solution-injected group (Leu group) and 12.0 g/L L-arginine solution-injected group (Arg group), amino acid was dissolved in 0.75% NaCl diluent solution. The Arg dosage was selected based on previous experimental results [32,33,34]. The Met and Leu dosages were calculated based on the amino acid balance in eggs [1]. The solution was freshly prepared and heated to 37.8 °C before injection. On day 17.5 of incubation, the amniotic cavity position was determined through candling. A 1 mm-diameter hole was drilled at the air cell end of the egg, and 0.5 mL of solution was injected into the amniotic cavity using a sterilized syringe with an 8-gauge needle. The hole was immediately sealed with paraffin wax. All injected eggs were then transferred to hatching baskets for continued incubation until day 21.

### 2.2. Broiler Rearing

On the day of hatch, chick sex was determined through feather sexing. Eight healthy male chicks were selected from each replicate and transferred to the poultry house at Changping Experimental Base of Chinese Academy of Agricultural Sciences (Beijing, China). Chicks were allowed free access to feed and water and other husbandry management practices were conducted in accordance with the AA Broiler Husbandry Manual (NY/T 33-2004) [35]. A corn-soybean meal diet was formulated based on the Brazilian standards [36], formulations and nutrient levels are shown in Table 1. The feeding trial lasted for 21 D.

### 2.3. Data and Sample Collection

At 19.5 days of incubation and 1, 7, and 21 days of age, one male broiler chicken closest to the average body weight of each replicate was euthanized by carbon dioxide method. Body weight, body length, tibia length, and pectoral muscle weight were recorded. Blood, liver, and ileum were collected. On the day of hatching, hatchability and rate of healthy chicks were calculated. At 7, 14, and 21 days of age, body weight and feed consumption per replicate were recorded. Average daily feed intake (ADFI), average daily gain (ADG), and feed conversion ratio (FCR) were calculated.

### 2.4. Serum Biochemistry

The levels of total protein (TP), albumin, urea nitrogen (BUN), uric acid (UA), triglycerides (TG), and total cholesterol (TC) in serum were determined. The kit was purchased from Jiancheng Bioengineering Institute, Nanjing, China.

### 2.5. Oxidation Indicator

The levels of catalase (CAT), glutathione peroxidase (GSH-Px), total superoxide dismutase (SOD), and malondialdehyde (MDA) in serum were determined. The kit was purchased from Jiancheng Bioengineering Institute, Nanjing, China.

### 2.6. Determination of Pro-Inflammatory Cytokine Concentrations

The levels of interleukin-1β (IL-1β), interleukin-8 (IL-8), and tumor necrosis factor-α (TNF-α) in the liver and ileum were determined. The kit was purchased from Shanghai Jianglai Biotechnology Co., Ltd., Shanghai, China.

### 2.7. Detection of mRNA Expression Level by qRT-PCR

Total RNA was extracted from liver and ileum tissue samples of broiler chickens using a fully automated RNA extraction system along with its supporting RNA extraction kit. RNA was reverse-transcribed to cDNA of mRNA using a reverse transcription kit. Gene expression was detected by the Real-Time Quantitative Fluorescence PCR Instrument (Bio-Rad, Hercules, CA, USA). RNA extraction kit, reverse transcription kit and PCR kit were purchased from Vazyme Biotech Co., Ltd., Nanjing, China. The coding DNA sequences of all genes were obtained from the NCBI website, and the corresponding primers were synthesized by Tsingke Biotech Co., Ltd., Beijing, China. All primers used in the present study are shown in Table 2. GAPDH was used as the internal reference gene. The relative expression levels of genes were calculated using the comparative 2−ΔΔCT method, and data analysis was performed using the Kruskal–Wallis (K-W) test in Prism v9.5 software.

### 2.8. Statistical Analyses

Statistical analysis was performed using the SPSS 26.0 software package. Data underwent normality tests and homogeneity of variance tests, followed by one-way ANOVA and Duncan’s multiple range tests (*p* < 0.05 was considered a significant difference). Results are expressed as mean ± standard error of the mean (X ± SEM).

## 3. Results

### 3.1. Embryo Development and Hatching Performance of Eggs

Compared to the NC group, IOF of Met significantly improved (*p* < 0.05) the hatchability. Conversely, the hatchability of the Leu group was significantly lower (*p* < 0.05) compared to the NC group (Table 3).

### 3.2. Growth Performance

The Met group exhibited significantly higher ADFI and ADG (*p* < 0.05) compared to the NC group during both the 8–21-day and 1–21-day experimental periods (Table 4).

The BL of the Met group at 1 d and the BW of the Met group at 21 d were significantly higher (*p* < 0.05) than those of the NC group (Table 5).

### 3.3. Serum Biochemistry

The BUN levels in both the Leu and Met groups were significantly reduced compared to the NC group at both 1 d and 21 d (*p* < 0.05). In contrast, the Arg group showed significantly lower BUN levels only at 1 d (*p* < 0.05) (Table 6).

### 3.4. Oxidation Indicator

Compared to the NC group, IOF of Arg significantly increased GSH-Px content at 1 d and CAT content at 21 d (*p* < 0.05). In contrast, IOF of Leu significantly increased CAT content at 21 d but decreased GSH-Px content at 1 d compared to those of the NC group (*p* < 0.05). More notably, compared to the NC group, IOF of Met significantly increased CAT content at 1 d and 21 d and GSH-Px content at 1 d (*p* < 0.05). Meanwhile, the CAT content of the MDA group at 1 d and 21 d was significantly lower than all other experimental groups (*p* < 0.05) (Table 7).

### 3.5. Inflammatory Cytokine Concentration

Compared to the NC group, IOF of Leu significantly decreased IL-8 concentration in the ileum at 21 d (*p* < 0.05), and IOF of Arg significantly decreased IL-8 concentration in the liver at 1 d and the ileum at 21 d (*p* < 0.05). Conversely, compared to the NC group, IOF of Met not only significantly decreased IL-8 and TNF-α concentration in the liver at 1 d and ileum at 1 and 21 d (*p* < 0.05) but also significantly decreased IL-1β concentration in the ileum at 1 d (*p* < 0.05) (Table 8 and Table 9).

### 3.6. Inflammatory Cytokine Concentration

**Figure 2:**
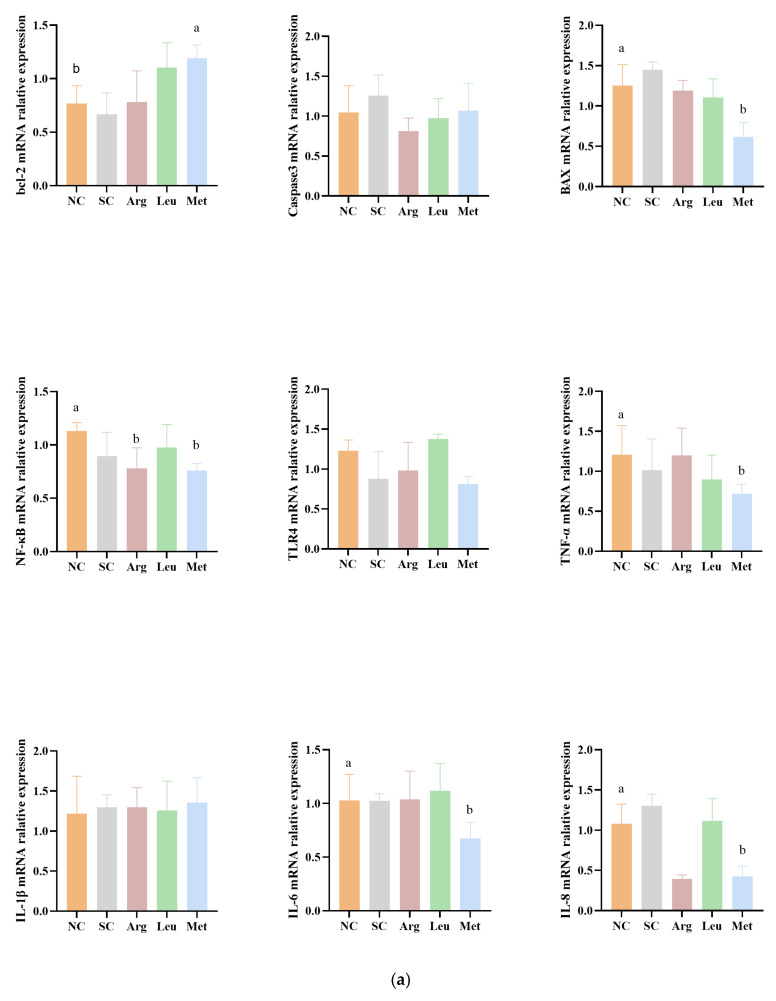

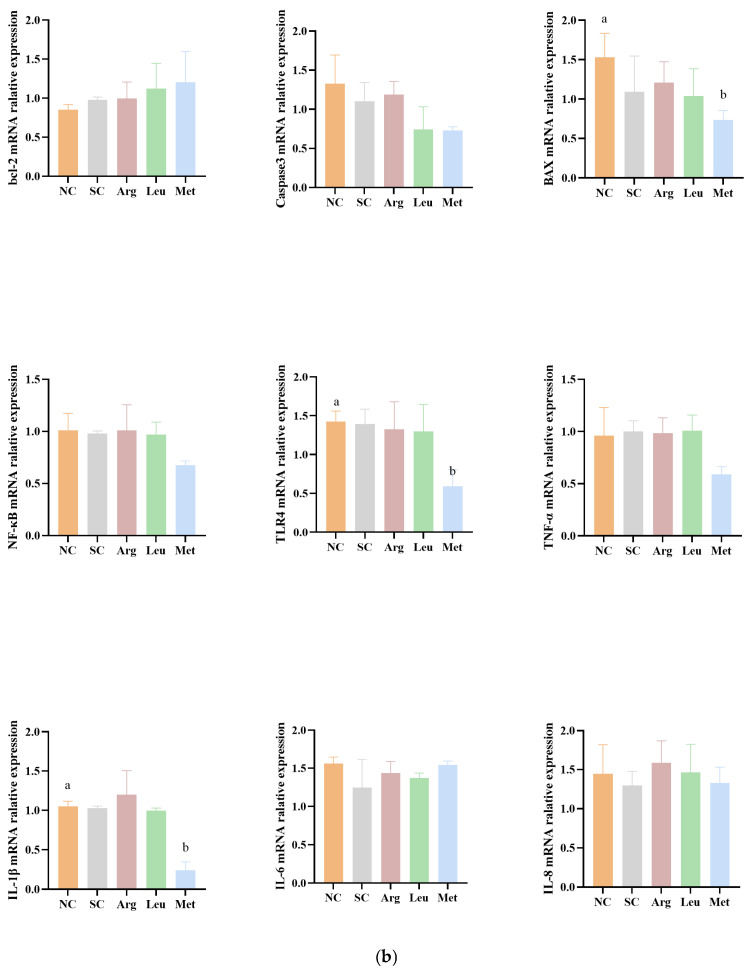

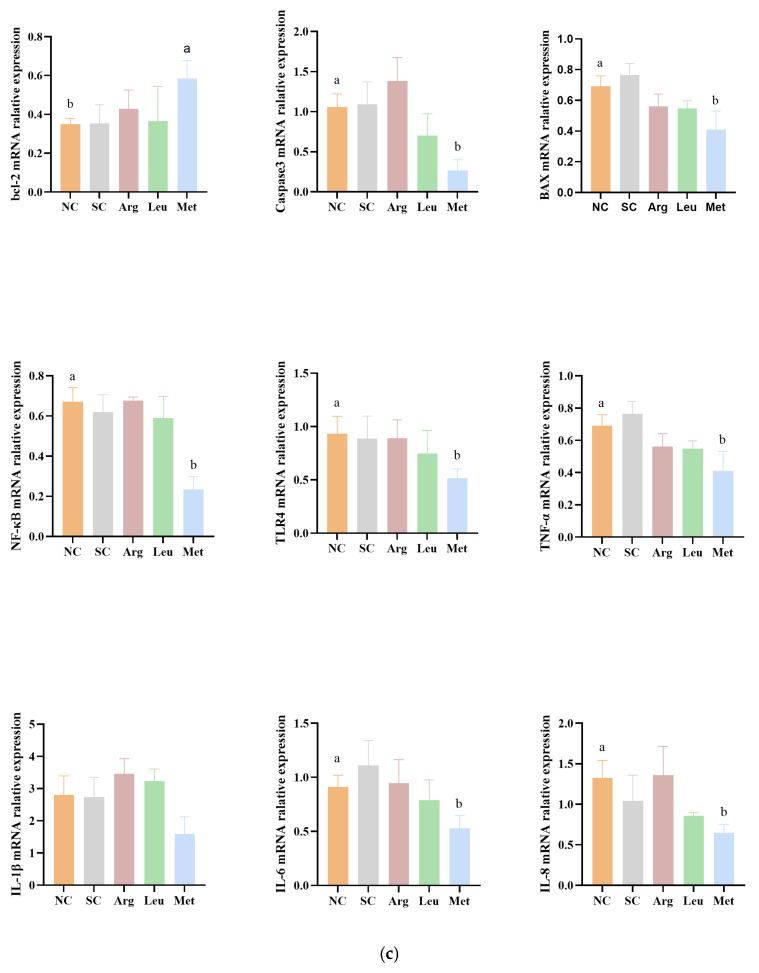

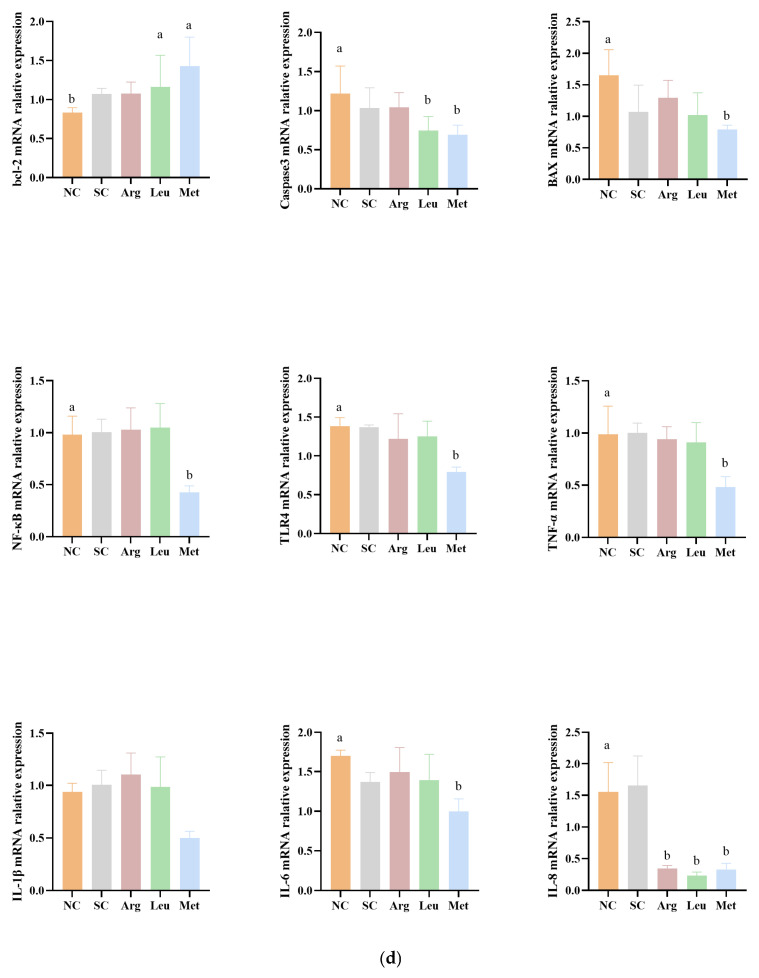
Effects of in ovo feeding Arg, Met, and Leu on mRNA relative expression levels of key genes involved in apoptosis and inflammatory pathways in liver and ileum of broiler chicken. In the figure, a and b represent statistically significant. (**a**) One-day-old liver tissue; (**b**) one-day-old ileum tissue; (**c**) 21-day-old liver tissue; (**d**) 21-day-old ileum tissue.

The mRNA relative expression levels of key genes involved in apoptosis and inflammatory pathways in the liver and ileum at 1 and 21 d are shown in Figure 2.

In the figure (a), compared to the NC group, IOF of Arg significantly decreased NF-κB relative expression in the liver at 1 d (*p* < 0.05). Conversely, IOF of Met not only significantly decreased BAX, NF-κB, TNF-α, IL-6, and IL-8 mRNA relative expression in the liver at 1 d (*p* < 0.05) but also significantly increased Bcl-2 mRNA relative expression (*p* < 0.05).

In the figure (b), compared to the NC group, IOF of Met significantly decreased BAX, TLR4, and IL-1β mRNA relative expression in the ileum at 1 d (*p* < 0.05).

In the figure (c), compared to the NC group, IOF of Met not only significantly decreased Caspase 3, BAX, NF-κB, TLR4, TNF-α, IL-6 and IL-8 mRNA relative expression in the liver at 21 d (*p* < 0.05) but also significantly increased Bcl-2 mRNA relative expression (*p* < 0.05).

In the figure (d), compared to the NC group, IOF of Arg only significantly decreased IL-8 mRNA relative expression in the ileum at 21 d (*p* < 0.05); IOF of Leu significantly decreased Caspase 3 and IL-8 mRNA relative expression and significantly increased Bcl-2 mRNA relative expression in the ileum at 21 d (*p* < 0.05); IOF of Met significantly decreased Caspase 3, BAX, NF-κB, TLR4, TNF-α, IL-6, and IL-8 mRNA relative expression in the ileum at 21 d (*p* < 0.05), but also significantly increased Bcl-2 mRNA relative expression (*p* < 0.05).

## 4. Discussion

### 4.1. Serum Biochemistry

Urea nitrogen is the main end product of protein metabolism in the body [37]. Oliva et al. [38] pointed out that the concentration of serum urea nitrogen can reflect the protein metabolism and the balance of amino acids in animals more accurately. The BUN levels of the Met group reduced, which suggests that Met enhanced the rate of protein anabolism over catabolism in chicks. As the first limiting amino acid in chicken, Met deficiency or excess disrupts amino acid homeostasis. This imbalance leads to deamination and degradation of unutilized amino acids into urea, consequently resulting in elevated blood urea levels.

### 4.2. Oxidation-Inflammation-Apoptosis

High temperatures readily induce oxidative stress in broilers [39], which is often accompanied by elevated inflammatory levels and the upregulated expression of apoptotic genes [40,41]. These processes collectively induce tissue damage, predispose to various diseases, and impair growth performance in broilers [42]. In this study, concurrent assessments of oxidative, inflammatory, and apoptotic markers revealed that the Met group demonstrated significant reductions across all three parameters. In contrast, no substantial differences were observed in the Arg and Leu groups compared to controls.

Under normal physiological conditions, an enzymatic system composed of GSH-Px, T-SOD, and CAT, as well as a non-enzymatic system, can clear active oxygen free radicals and reduce lipid peroxidation reactions through an enzymatic and non-enzymatic system, thus protecting the body from oxidative stress [43]. MDA is the end product of lipid peroxidation, and its content can reflect the oxidative state of the body [44]. In this study, the content of antioxidant enzymes in all treatment groups increased, but only the MDA content in the Met group significantly decreased. It can be seen that IOF of Met can enhance the antioxidant capacity of chicken embryos. Metabolic mechanism analysis suggests that methionine’s antioxidant effects may be closely associated with its biological characteristics as a sulfur-containing amino acid (SAA). Firstly, Met serves as a precursor for cysteine, modulating oxidative stress responses by influencing cellular redox status [45]. Secondly, its metabolites—S-adenosylmethionine, polyamines, and glutathione (GSH)—play pivotal roles in antioxidant defense systems [46].

IL-1β, IL-8, and TNF-α are key pro-inflammatory cytokines involved in inflammatory and immune responses. These cytokines induce cellular apoptosis and inflammatory cascades, with elevated concentrations observed under oxidative stress conditions [47]. In this experiment, in ovo Met supplementation not only significantly reduced IL-8 and TNF-α concentrations in the liver of 1-day-old chicks and the ileum of both 1-day-old and 21-day-old chicks but also markedly decreased IL-1β levels in the ileum of 1-day-old chicks. Notably, the anti-inflammatory effects exhibited sustained predominance in intestinal tissues, whereas hepatic anti-inflammatory responses showed age-dependent attenuation. This suggests temporal differences in nutrient responsiveness across organs. Mechanistically corroborating the observed reductions in pro-inflammatory cytokine concentrations, the Met group also demonstrated significant downregulation of pro-inflammatory transcriptional levels. Concurrently, mRNA expression of TLR4 and its downstream NF-κB was inhibited. TLR4 activates NF-κB by recognizing endogenous damage-associated molecular patterns (DAMPs) [48], while nuclear translocation of NF-κB drives excessive pro-inflammatory cytokine expression [47]. This study reveals that Met may suppress transcriptional activation of inflammation-related genes by blocking the TLR4-NF-κB signaling cascade. These findings provide reverse validation with Peng et al.’s [49] research on Met deficiency exacerbating hepatic inflammatory factor expression, collectively confirming the anti-inflammatory properties of Met.

Bcl-2, an anti-apoptotic protein, sustains cell survival by suppressing BAX activity [50]. This study demonstrated that IOF of Met significantly upregulated Bcl-2 mRNA expression while downregulating BAX and Caspase 3 levels. Mechanistically, Bcl-2 may inhibit BAX-mediated mitochondrial cytochrome c release [51], potentially blocking Caspase 3 activation during the apoptotic execution phase to maintain cellular survival rates [52].

### 4.3. Hatching Performance and Growth Development

In this study, in ovo injection of three amino acids showed no significant effects on embryonic development or the rate of healthy chicks, but markedly influenced hatchability. Concurrently, in ovo Met administration was found to promote post-hatch growth and development. Unlike previous studies demonstrating significant increases in 1 D BW/SEW, EW, and EW/SEW through amino acid injection [1,34], this experiment revealed divergent outcomes. These discrepancies may be attributed to variations in solution concentration, injection timing/site [53], or amino acid configurations. For fertilized breeding eggs, hatchability remains a paramount indicator. This study observed no significant impact of Arg injection on hatchability, whereas Leu injection significantly reduced hatchability, and Met injection significantly improved it. The Arg group results align with findings from Foye et al. [16] and Tangara et al. [11]. Notably, Farias et al. [54] reported decreased hatchability after injecting five Met concentrations into the amniotic cavity on day 16 of incubation, with concentrations exceeding 1% causing more pronounced reductions. This underscores the critical need for precise Met concentration control, as excessive levels may induce adverse effects. Kop-Bozbay et al. [55] further corroborated these findings by demonstrating significant hatchability decline after injecting a branched-chain amino acid mixture (L-leucine, L-valine, L-isoleucine) into turkey amniotic cavities on day 22 of incubation, consistent with the current experimental results.

The results of this study showed that the ADFI and ADG of the Met group and the BW of the Met group at Day 21 were significantly increased. It is not difficult to infer that intra-ovarian injection of Met improved ADFI of chicks, thereby improving ADG. According to Nazem et al. [7] and Elwan et al. [56] this increase in body weight of chicks at hatch submitted to IOF of Met may be attributed to the improved antioxidant status of the embryos.

Elwan et al. [56] also reported that since Met metabolism affects oxidative status, it is reasonable to hypothesize that dietary Met supplementation may play a beneficial role in alleviating oxidative stress and, consequently, provide better conditions for embryonic development at the final stage. Integrating the observed reductions in serum BUN levels and significant declines in oxidative, inflammatory, and apoptotic markers in the Met group, we hypothesize that Met maintains broiler developmental homeostasis by establishing an antioxidant-anti-inflammatory-anti-apoptotic synergistic defense system. Firstly, Met significantly enhances antioxidant enzyme activity and reduces MDA levels, effectively mitigating oxidative stress induced by summer heat. Secondly, Met suppresses transcriptional activation of inflammatory cytokines by inhibiting the TLR4-NF-κB pathway, thereby lowering systemic inflammation in broilers. Additionally, Met upregulates the expression of the anti-apoptotic protein Bcl-2 while inhibiting transcriptional activation of apoptotic proteins such as Caspase 3, thereby preserving cellular survival rates. Notably, Met also promotes protein synthesis and downregulates serum BUN levels. This study represents the first systematic exploration of the mechanisms by which in ovo Met supplementation improves broiler growth and development through multidimensional regulatory networks. By elucidating its biological mechanisms at molecular, cellular, and tissue levels, we provide both theoretical foundations and practical strategies for precise nutritional regulation in poultry production.

### 4.4. Limitation

This experiment assessed male broilers only during the 1–21 day period. Future studies should extend observations to additional developmental stages and include female broilers for further investigation.

## 5. Conclusions

Overall, the injection of arginine and leucine into chicken embryos had no significant effect, while supplementation of Met to chicken embryos could promote the growth and development of chicks, promote protein synthesis, and enhance the antioxidant and anti-inflammatory abilities of the body.

Currently, in ovo injection technology has not achieved widespread adoption in livestock production. By integrating early nutritional supplementation with commercial vaccine diluents through automated inovoject (capable of processing 20,000–30,000 hatching eggs per hour), this approach may potentially reduce operational costs and accelerate the commercialization of in-ovo injection techniques.

## Figures and Tables

**Table 1:** Composition and nutrient levels of the basal diet.

Items	Content	
	1–7 Days	8–21 Days
Ingredients (%)		
Corn	51.44	54.08
Soybean meal	40.21	36.82
Soybean oil	3.94	5.00
Limestone	1.00	0.85
CaHPO_4_	1.89	1.80
NaCl	0.30	0.30
DL-Methionine	0.21	0.19
L-Lysine	0.36	0.32
L-Threonine	0.15	0.14
premix ^1^	0.50	0.50
Total	100	100
Nutrient levels ^2^		
ME, Kcal/kg	2961	3038
CP (%)	22. 55	21.18
EE(%)	10.54	10.38
Ca (%)	0.94	0.85
AP (%)	0.43	0.41
L-Lysine (%)	1.46	1.34
DL-Methionine (%)	0.54	0.51
DL-Methionine + L-Cysteine (%)	0.92	0.87

Abbreviations: ME, metabolizable energy; CP, crude protein; AP, available phosphorus; EE, ether extract. ^1^ Premix provided the following per kg of the diet: 1–7 d: vitamin A 12,000 IU, vitamin D3 5000 IU, vitamin E 80 mg, vitamin K3 3.2 mg, vitamin B1 3.2 mg, vitamin B2 8.6 mg, vitamin B6 4.3 mg, vitamin B12 17 g, pantothenic acid calcium 20 mg, nicotinic acid 65 mg, folic acid 2.2 mg, biotin 0.22 mg, choline 1020 mg, Cu (CuSO_4_·5H_2_O) 16 mg, Fe (FeSO_4_·7H_2_O) 20 mg, Zn (ZnSO_4_·7H_2_O) 110 mg, Mn (MnSO_4_·H_2_O) 120 mg, Se (Na_2_SeO_3_) 0.3 mg, and I (KI) 1.25 mg; 8–21 d: vitamin A 10,000 IU, vitamin D3 4500 IU, vitamin E 65 mg, vitamin K3 3.0 mg, vitamin B1 2.5 mg, vitamin B2 6.5 mg, vitamin B6 3.2 mg, vitamin B12 17 g, pantothenic acid calcium 18 mg, nicotinic acid 60 mg, folic acid 1.9 mg, biotin 0.18 mg, choline 1020 mg, Cu (CuSO_4_·5H_2_O) 16 mg, Fe (FeSO_4_·7H_2_O) 20 mg, Zn (ZnSO_4_·7H_2_O) 110 mg, Mn (MnSO_4_·H_2_O) 120 mg, Se (Na_2_SeO_3_) 0.3 mg, and I (KI) 1.25 mg; ^2^ nutrient levels were measured value.

**Table 2:** Gene primer information.

Gene Name	Gene Sequence
BAX	F: TGAGCATGTAGCAACGGAAG
	R: AGCAAGCTGATTGACGGTCT
Caspase 3	F: CATCT GCATCC GTGCCTGA
	R: CTCTCGG CTGTGGTGGTGAA
Bcl-2	F: AGAGGGACTTCGCCCAGAT
	R: AGGCATCCCATCCTCCGT
TLR4	F: AGT CTG AAA TTG CTG AGC TCA AAT
	R: GCG ACG TTA AGC CAT GGA AG
NF-κB	F: TACTGATTGCTGCTGGAGTTGATGTC
	R: TTGTGCCATCGTATGTAGTGCTGTC
IL-1β	F: CTGCCTGCAGAAGAAGCCT
	R: TCAGGCATTTCTCCTCGTCG
IL-6	F: CTGCTGCCGCTGCTGCTG
	R: TCTCGCACACGGTGAACTTCTTG
IL-8	F: GCTCTGTCGCAAGGTAGGACG
	R: TCACAGTGGTGCATCAGAATT
TNF-α	F: GGACAGCCTATGCCAACAAG
	R: ACACGACAGCCAAGTCAACG
GAPDH	F: GACAGCCATTCCTCCACCTT
	R: TGCCATGTGGACCATCAAGT

**Table 3:** Effects of in ovo feeding Arg, Met, and Leu on embryo development and hatching performance of eggs.

	Treatments					
Items	NC	SC	Arg	Leu	Met	*p-*Value
SEW (g)	62.69 ± 0.55	63.90 ± 1.66	64.97 ± 1.28	65.48 ± 1.04	63.24 ± 0.81	0.403
EW (g)	55.19 ± 0.77	55.83 ± 1.71	57.68 ± 1.19	58.08 ± 1.09	56.92 ± 0.92	0.393
19.5 D BW (g)	35.52 ± 0.91	33.56 ± 1.28	34.42 ± 2.42	32.99 ± 0.90	35.17 ± 0.96	0.691
19.5 D YSW (g)	9.37 ± 0.53	10.84 ± 0.49	10.69 ± 74	12.16 ± 1.06	10.67 ± 0.47	0.123
1 D BW (g)	45.58 ± 1.43	47.14 ± 1.76	47.95 ± 1.50	50.07 ± 0.70	46.82 ± 1.50	0.274
EW/SEW	88.02 ± 0.77	87.33 ± 0.89	88.79 ± 0.47	88.69 ± 0.72	89.99 ± 0.61	0.132
1 D BW/SEW	56.63 ± 1.12	52.58 ± 1.82	52.86 ± 3.25	50.34 ± 0.72	55.57 ± 0.99	0.142
19.5 D YSW/19.5 D BW	26.63 ± 2.06	32.46 ± 1.77	31.90 ± 3.09	37.31 ± 4.12	30.52 ± 1.79	0.127
Hatchability (%)	84.60 ± 1.19 ^b^	84.64 ± 1.15 ^b^	84.59 ± 1.27 ^b^	79.14 ± 1.54 ^c^	89.54 ± 1.63 ^a^	0.001
Rate of healthy chicks (%)	93.96 ± 1.21	95.48 ± 1.43	94.21 ± 1.16	97.62 ± 1.51	96.88 ± 1.40	0.255

^a–c^ Means within a row without common superscript differ significantly (*p* < 0.05). N = 6, one embryo per replicate. NC: non-punctured control group. SC: saline-injected control group. Arg: Arg solution-injected group. Leu: Leu solution-injected group. Met: Met solution-injected group. SEW: set egg weight. EW: egg weight at 19.5 day. BW: body weight. YSW: yolk sac weight.

**Table 4:** Effects of in ovo feeding Arg, Met, and Leu on ADFI, ADG, and FCR of broiler chicken.

	Treatments					
Items	NC	SC	Arg	Leu	Met	*p-*Value
1–7 d						
ADFI (g)	17.82 ± 0.41	18.03 ± 0.31	16.99 ± 0.56	17.54 ± 0.51	17.95 ± 0.57	0.564
ADG (g)	17.00 ± 0.37	17.57 ± 0.35	16.28 ± 0.58	16.99 ± 0.59	17.11 ± 0.60	0.529
FCR (g/g)	1.05 ± 0.01	1.03 ± 0.00	1.04 ± 0.01	1.03 ± 0.01	1.05 ± 0.01	0.293
8–21 d						
ADFI (g)	60.14 ± 0.82 ^b^	59.40 ± 2.79 ^b^	59.75 ± 0.91 ^b^	59.46 ± 0.94 ^b^	66.03 ± 1.26 ^a^	0.022
ADG (g)	45.53 ± 0.66 ^b^	44.31 ± 1.94 ^b^	45.43 ± 0.81 ^b^	45.6 ± 1.27 ^b^	50.99 ± 0.93 ^a^	0.006
FCR (g/g)	1.32 ± 0.02	1.34 ± 0.03	1.32 ± 0.03	1.31 ± 0.03	1.30 ± 0.04	0.830
1–21 d						
ADFI (g)	40.58 ± 1.00 ^b^	39.77 ± 1.06 ^b^	40.17 ± 0.42 ^b^	40.98 ± 1.10 ^b^	44.20 ± 1.24 ^a^	0.034
ADG (g)	37.13 ± 1.52 ^b^	35.40 ± 1.27 ^b^	35.71 ± 0.54 ^b^	36.07 ± 0.82 ^b^	40.38 ± 1.30 ^a^	0.031
FCR (g/g)	1.10 ± 0.03	1.13 ± 0.04	1.13 ± 0.01	1.14 ± 0.02	1.10 ± 0.02	0.685

^a,b^ Means within a row without common superscript differ significantly (*p* < 0.05). N = 6. NC: non-punctured control group. SC: saline-injected control group. Arg: Arg solution-injected group. Leu: Leu solution-injected group. Met: Met solution-injected group. ADFI: average daily food intake. ADG: Average daily weight gain. FCR: feed conversion ratio.

**Table 5:** Effects of in ovo feeding Arg, Met, and Leu on the body weight, body length, tibial length, and pectoral muscle weight of chick embryos at 19.5 d of incubation and post-hatch broiler chicken.

	Treatments					
Items	NC	SC	Arg	Leu	Met	*p-*Value
At 19.5 d of incubation						
BW (g)	35.52 ± 0.91	33.56 ± 1.28	34.42 ± 2.42	32.99 ± 0.90	35.17 ± 0.96	0.691
BL (mm)	81.29 ± 2.11	82.01 ± 2.87	85.98 ± 1.13	87.05 ± 1.34	86.21 ± 1.30	0.123
TL (mm)	24.14 ± 0.27	23.62 ± 0.51	23.64 ± 0.37	23.70 ± 0.27	23.75 ± 0.58	0.901
PMW (g)	0.27 ± 0.01	0.27 ± 0.01	0.28 ± 0.06	0.27 ± 0.02	0.29 ± 0.01	0.796
At 1 d						
BW (g)	45.58 ± 1.43	47.14 ± 1.76	47.95 ± 1.50	50.07 ± 0.70	46.82 ± 1.50	0.274
BL (mm)	96.82 ± 0.98 ^b^	96.09 ± 1.29 ^b^	96.94 ± 1.51 ^b^	93.83 ± 0.84 ^b^	100.97 ± 0.97 ^a^	0.004
TL (mm)	22.40 ± 0.68	22.53 ± 0.56	22.97 ± 0.74	22.45 ± 0.52	23.10 ± 0.82	0.921
PMW (g)	0.31 ± 0.02	0.31 ± 0.02	0.31 ± 0.03	0.32 ± 0.02	0.31 ± 0.01	0.996
At 7 d						
BW (g)	180.52 ± 3.29	192.18 ± 3.49	183.49 ± 7.04	192.62 ± 7.44	188.12 ± 5.58	0.484
BL (mm)	147.03 ± 1.42	147.84 ± 2.60	141.53 ± 8.41	153.35 ± 1.18	148.83 ± 2.03	0.400
TL (mm)	34.73 ± 0.84	34.18 ± 0.62	33.65 ± 0.53	35.41 ± 0.28	35.23 ± 0.37	0.185
PMW (g)	5.94 ± 0.18	5.96 ± 0.27	5.94 ± 0.44	6.06 ± 0.28	6.30 ± 0.56	0.953
At 21 d						
BW (g)	800.72 ± 36.94 ^b^	834.10 ± 22.07 ^b^	850.17 ± 25.32 ^b^	828.33 ± 14.01 ^b^	939.00 ± 31.28 ^a^	0.016
BL (cm)	23.83 ± 0.36	23.70 ± 0.38	23.50 ± 0.45	24.05 ± 0.22	24.92 ± 0.39	0.093
TL (mm)	58.18 ± 0.49	56.51 ± 0.31	56.22 ± 1.39	57.68 ± 1.36	58.37 ± 0.97	0.459
PMW (g)	64.36 ± 5.38	70.22 ± 1.69	75.17 ± 4.76	66.20 ± 1.71	80.04 ± 5.56	0.081

^a,b^ Means within a row without common superscript differ significantly (*p* < 0.05). N = 6, one chick per replicate. NC: non-punctured control group. SC: saline-injected control group. Arg: Arg solution-injected group. Leu: Leu solution-injected group. Met: Met solution-injected group. BW: body weight. BL: body length. TL: tibial length. PMW: pectoral muscle weight.

**Table 6:** Effects of in ovo feeding Arg, Met, and Leu on serum biochemical index of broiler chicken.

	Treatments					
Items	NC	SC	Arg	Leu	Met	*p-*Value
At 1 d						
TP (g/L)	17.65 ± 45.00	19.76 ± 13.78	21.32 ± 16.43	23.72 ± 34.44	27.03 ± 18.84	0.197
ALB (g/L)	12.52 ± 0.56	13.82 ± 1.38	15.57 ± 1.68	14.11 ± 1.13	16.62 ± 1.27	0.205
TC (mmol/L)	12.07 ± 1.00	13.95 ± 1.68	12.30 ± 0.49	12.11 ± 1.21	13.90 ± 0.52	0.533
TG (mmol/L)	1.12 ± 0.17	1.31 ± 0.09	1.15 ± 0.08	1.11 ± 0.13	1.50 ± 0.14	0.166
UA (U/L)	335.27 ± 54.48	473.07 ± 73.16	522.95 ± 40.52	442.63 ± 57.75	543.72 ± 30.03	0.080
BUN (mmol/L)	1.12 ± 0.14 ^a^	1.01 ± 0.11 ^a^	0.66 ± 0.15 ^b^	0.61 ± 0.10 ^b^	0.62 ± 0.02 ^b^	0.023
At 21 d						
TP (g/L)	31.92 ± 1.28	36.93 ± 1.92	34.88 ± 4.24	32.14 ± 2.22	34.94 ± 3.60	0.706
ALB (g/L)	13.31 ± 0.25	13.16 ± 1.33	11.08 ± 1.12	16.24 ± 2.65	12.22 ± 0.98	0.190
TC (mmol/L)	4.61 ± 0.88	4.71 ± 0.45	2.76 ± 0.15	4.40 ± 1.30	2.82 ± 0.22	0.165
TG (mmol/L)	0.50 ± 0.07	0.51 ± 0.06	0.75 ± 0.16	0.45 ± 0.03	0.49 ± 0.12	0.249
UA (U/L)	264.96 ± 31.03	247.38 ± 30.99	246.89 ± 38.76	227.41 ± 22.02	270.03 ± 38.61	0.898
BUN (mmol/L)	0.32 ± 0.05 ^a^	0.31 ± 0.03 ^a^	0.33 ± 0.06 ^a^	0.22 ± 0.02 ^b^	0.15 ± 0.01 ^c^	0.012

^a–c^ Means within a row without common superscripts differ significantly (*p* < 0.05). N = 6, one chick per replicate. NC: non-punctured control group. SC: saline-injected control group. Arg: Arg solution-injected group. Leu: Leu solution-injected group. Met: Met solution-injected group. TP: Total protein. ALB: Albumin. TC: Total cholesterol. TG: Triglycerides. UA: Uric acid. BUN: Urea nitrogen.

**Table 7:** Effects of in ovo feeding Arg, Met, and Leu on oxidation index of broiler chicken.

	Treatments					
Items	NC	SC	Arg	Leu	Met	*p-*Value
At 1 d						
CAT/(U/mL)	6.51 ± 0.89 ^b^	5.99 ± 0.38 ^b^	7.09 ± 0.36 ^b^	6.74 ± 0.55 ^b^	12.86 ± 0.89 ^a^	<0.001
GSH-Px/(U/mL)	443.02 ± 18.83 ^b^	441.51 ± 20.94 ^b^	509.89 ± 29.27 ^a^	397.04 ± 26.55 ^c^	492.33 ± 24.75 ^a^	0.024
SOD/(U/mL)	76.54 ± 4.11	82.18 ± 5.09	94.22 ± 5.67	96.20 ± 5.71	89.05 ± 3.39	0.082
MDA/(nmol/mL)	7.52 ± 0.61 ^a^	7.48 ± 0.57 ^a^	6.40 ± 0.65 ^a^	6.59 ± 0.29 ^a^	4.66 ± 0.34 ^b^	0.004
At 21 d						
CAT/(U/mL)	3.34 ± 0.62 ^b^	3.33 ± 0.65 ^b^	8.74 ± 0.46 ^a^	8.40 ± 0.65 ^a^	7.63 ± 1.55 ^a^	0.002
GSH-Px/(U/mL)	600.75 ± 35.01	640.75 ± 13.52	631.70 ± 22.54	607.55 ± 8.70	592.45 ± 13.20	0.433
SOD/(U/mL)	121.36 ± 1.65	120.34 ± 1.81	119.91 ± 2.00	120.90 ± 1.89	122.57 ± 1.78	0.865
MDA/(nmol/mL)	4.23 ± 0.47 ^a^	3.94 ± 0.47 ^a^	3.50 ± 0.33 ^a^	3.34 ± 0.27 ^a^	2.20 ± 0.35 ^b^	0.011

^a–c^ Means within a row without common superscripts differ significantly (*p* < 0.05). N = 6, one chick per replicate. NC: non-punctured control group. SC: saline-injected control group. Arg: Arg solution-injected group. Leu: Leu solution-injected group. Met: Met solution-injected group. CAT: catalase. GSH-Px: glutathione peroxidase. SOD: superoxide dismutase. MDA: malondialdehyde.

**Table 8:** Effects of in ovo feeding Arg, Met, and Leu on the Concentration of Inflammatory Cytokines in the liver and ileum of 1-day-old broiler chicken.

	Treatments					
Items	NC	SC	Arg	Leu	Met	*p-*Value
Liver						
IL-1β/(pg/mL)	0.4 ± 0.08	0.50 ± 0.10	0.39 ± 0.05	0.37 ± 0.05	0.31 ± 0.04	0.413
IL-8/(pg/mL)	13.35 ± 0.38 ^a^	13.20 ± 0.59 ^a^	12.43 ± 0.52 ^b^	13.30 ± 0.90 ^a^	10.68 ± 0.56 ^b^	0.023
TNF-α/(pg/mL)	10.57 ± 0.57 ^a^	10.00 ± 0.65 ^a^	12.00 ± 0.78 ^a^	10.74 ± 0.49 ^a^	8.10 ± 0.70 ^b^	0.015
Ileum						
IL-1β/(pg/mL)	0.61 ± 0.04 ^a^	0.65 ± 0.02 ^a^	0.64 ± 0.02 ^a^	0.58 ± 0.04 ^ab^	0.50 ± 0.03 ^b^	0.023
IL-8/(pg/mL)	15.72 ± 0.67	15.52 ± 0.50	15.48 ± 0.71	14.94 ± 0.57	13.31 ± 0.84	1.102
TNF-α/(pg/mL)	14.11 ± 0.71	13.97 ± 0.58	12.73 ± 0.69	13.53 ± 0.76	11.55 ± 0.64	0.075

^a,b^ Means within a row without common superscript differ significantly (*p* < 0.05). N = 6, one chick per replicate. NC: non-punctured control group. SC: saline-injected control group. Arg: Arg solution-injected group. Leu: Leu solution-injected group. Met: Met solution-injected group.

**Table 9:** Effects of in ovo feeding Arg, Met, and Leu on the Concentration of Inflammatory Cytokines in the liver and ileum of 21-day-old broiler chicken.

	Treatments					
Items	NC	SC	Arg	Leu	Met	*p-*Value
Liver						
IL-1β/(pg/mL)	2.83 ± 0.32	2.74 ± 0.24	3.08 ± 0.43	2.09 ± 0.15	2.08 ± 0.25	0.093
IL-8/(pg/mL)	14.61 ± 0.73 ^a^	13.76 ± 0.21 ^a^	13.91 ± 0.55 ^a^	13.27 ± 0.71 ^ab^	11.26 ± 0.68 ^b^	0.015
TNF-α/(pg/mL)	12.48 ± 1.12 ^a^	12.90 ± 0.92 ^a^	13.17 ± 0.87 ^a^	12.57 ± 0.70 ^a^	9.55 ± 0.61 ^b^	0.017
Ileum						
IL-1β/(pg/mL)	2.11 ± 0.32	2.14 ± 0.24	2.31 ± 0.43	2.96 ± 0.15	1.64 ± 0.25	0.409
IL-8/(pg/mL)	18.86 ± 0.73 ^a^	18.11 ± 0.21 ^a^	15.77 ± 0.55 ^b^	15.46 ± 0.71 ^b^	15.33 ± 0.68 ^b^	0.002
TNF-α/(pg/mL)	16.80 ± 1.12 ^a^	16.81 ± 0.92 ^a^	14.03 ± 0.87 ^ab^	16.01 ± 0.70 ^a^	12.26 ± 0.61 ^b^	0.007

^a,b^ Means within a row without common superscript differ significantly (*p* < 0.05). N = 6, one chick per replicate. NC: non-punctured control group. SC: saline-injected control group. Arg: Arg solution-injected group. Leu: Leu solution-injected group. Met: Met solution-injected group.

## Data Availability

The original contributions presented in this study are included in the article. Further inquiries can be directed to the corresponding author.

## References

[1] Ohta Y., Tsushima N., Koide K., Kidd M.T., Ishibashi T. (1999). Effect of amino acid injection in broiler breeder eggs on embryonic growth and hatchability of chicks. Poult. Sci..

[2] Oliveira J.E.D., Uni Z., Ferket P.R. (2008). Important metabolic pathways in poultry embryos prior to hatch. World’s Poult. Sci. J..

[3] Moran E.T., Reinhart B.S. (1980). Poult yolk sac amount and composition upon placement: Effect of breeder age, egg weight, sex, and subsequent change with feeding or fasting. Poult. Sci..

[4] Meng Y., Qiu N., Mine Y., Keast R. (2021). Comparative Lipidomics of Chick Yolk Sac during the Embryogenesis Provides Insight into Understanding the Development-Related Lipid Supply. J. Agric. Food Chem..

[5] Willemsen H., Debonne M., Swennen Q., Everaert N., Careghi C., Han H., Bruggeman V., Tona K., Decuypere E. (2010). Delay in feed access and spread of hatch: Importance of early nutrition. World’s Poult. Sci. J..

[6] Gao T., Zhao M.M., Zhang L., Li J.L., Yu L.L., Lv P.A., Gao F., Zhou G.H. (2017). Effects of in ovo feeding of l-arginine on the development of lymphoid organs and small intestinal immune barrier function in posthatch broilers. Anim. Feed Sci. Technol..

[7] Naser N.M., Mohsen S.S., Reza K., Hamideh M. (2017). Histomorphometric analysis of the small intestine of broiler chick embryos injected in ovo with methionine. Anim. Prod. Sci..

[8] Kadam M.M., Barekatain M.R., Bhanja S.K., Iji P.A. (2013). Prospects of in ovo feeding and nutrient supplementation for poultry: The science and commercial applications—A review. J. Sci. Food Agric..

[9] Foye O.T., Uni Z., Ferket P.R. (2006). Effect of in ovo feeding egg white protein, beta-hydroxy-beta-methylbutyrate, and carbohydrates on glycogen status and neonatal growth of turkeys. Poult. Sci..

[10] Foye O.T., Uni Z., Mcmurtry J.P., Ferket P.R. (2006). The Effects of Amniotic Nutrient Administration, “In ovo Feeding” of Arginine And/or β-Hydroxy- Betβ-Methyl Butyrate (HMB) on Insulin-like Growth Factors, Energy Metabolism and Growth in Turkey Poults. Int. J. Poult. Sci..

[11] Tangara M., Chen W., Xu J., Huang F.R., Peng J. (2010). Effects of in ovo feeding of carbohydrates and arginine on hatchability, body weight, energy metabolism and perinatal growth in duck embryos and neonates. Br. Poult. Sci..

[12] Pesti G.M. (1995). Nutrient requirements of poultry. Anim. Feed Sci. Technol..

[13] Kidd M. (2004). Nutritional modulation of immune function in broilers. Poult. Sci..

[14] Tesseraud S., Coustard S.M., Collin A., Seiliez I. (2009). Role of sulfur amino acids in controlling nutrient metabolism and cell functions: Implications for nutrition. Br. J. Nutr..

[15] Çoşkun İ., Erener G., Şahin A., Karadavut U., Okur A.A. (2014). Impacts of In Ovo Feeding of DL-Methionine on Hatchability and Chick Weight. Turk. J. Agric. Food Sci. Technol..

[16] Foye O.T., Ferket P.R., Uni Z. (2007). The effects of in ovo feeding arginine, beta-hydroxy-beta-methyl-butyrate, and protein on jejunal digestive and absorptive activity in embryonic and neonatal turkey poults. Poult. Sci..

[17] Kadam M.M., Bhanja S.K., Mandal A.B., Thakur R., Vasan P., Bhattacharyya A., Tyagi J.S. (2008). Effect of in ovo threonine supplementation on early growth, immunological responses and digestive enzyme activities in broiler chickens. Br. Poult. Sci..

[18] Kita K., Ito K.R., Sugahara M., Kobayashi M., Makino R., Takahashi N., Nakahara H., Takahashi K., Nishimukai M. (2015). Effect of In Ovo Administration of Branched-Chain Amino Acids on Embryo Growth and Hatching Time of Chickens. J. Poult. Sci..

[19] Han G., Yang H., Bungo T., Ikeda H., Chowdhury V.S. (2018). In ovo L-leucine administration stimulates lipid metabolisms in heat-exposed male, but not female, chicks to afford thermotolerance. J. Therm. Biol..

[20] Han G., Ouchi Y., Hirota T., Haraguchi S., Miyazaki T., Arakawa T., Masuhara N., Mizunoya W., Tatsumi R., Tashiro K. (2020). Effects ofl-leucinein ovofeeding on thermotolerance, growth and amino acid metabolism under heat stress in broilers. Animal.

[21] Han G., Yang H., Tashiro K., Bungo T., Furuse M., Chowdhury V.S. (2019). In ovo administration of L-leucine: A novel approach to affording thermotolerance in broiler chicks. J. Therm. Biol..

[22] Han G., Yang H., Bahry M.A., Tran P.V., Do P.H., Ikeda H., Furuse M., Chowdhury V.S. (2017). L-Leucine acts as a potential agent in reducing body temperature at hatching and affords thermotolerance in broiler chicks. Comp. Biochem. Physiol. Part A Mol. Integr. Physiol..

[23] Han G., Ren Y., Shen D., Li S., Chowdhury V.S., Li Y., Furuse M., Li C. (2021). L-leucine in ovo administration causes growth retardation and modifies specific amino acid metabolism in broiler embryos. J. Poult. Sci..

[24] Han G., Yang H., Wang Y., Zhang R., Tashiro K., Bungo T., Furuse M., Chowdhury V.S. (2019). Effects of in ovo feeding of L-leucine on amino acids metabolism and heat-shock protein-70, and -90 mRNA expression in heat-exposed chicks. Poult. Sci..

[25] Batool F., Bilal R.M., Hassan F.U., Nasir T.A., Rafeeque M., Elnesr S.S., Farag M.R., Mahgoub H.A.M., Naiel M.A.E., Alagawany M. (2023). An updated review on behavior of domestic quail with reference to the negative effect of heat stress. Anim. Biotechnol..

[26] Nawab A., Ibtisham F., Li G., Kieser B., Wu J., Liu W., Zhao Y., Nawab Y., Li K., Xiao M. (2018). Heat stress in poultry production: Mitigation strategies to overcome the future challenges facing the global poultry industry. J. Therm. Biol..

[27] El-Tarabany M.S., Nassan M.A., Salah A.S. (2021). Royal Jelly Improves the Morphology of the Reproductive Tract, Internal Egg Quality, and Blood Biochemical Parameters in Laying Hens at the Late Stage of Production. Animals.

[28] Park S.U., Walsh L., Berkowitz K.M. (2021). Mechanisms of ovarian aging. Reproduction.

[29] Lillpers K., Wilhelmson M. (1993). Age-Dependent Changes in Oviposition Pattern and Egg Production Traits in the Domestic Hen. Poult. Sci..

[30] Chen J., Wang Y., Tang Z., Guo X., Yuan J. (2023). Impact of Dietary Supplementation of Cysteamine on Egg Taurine Deposition, Egg Quality, Production Performance and Ovary Development in Laying Hens. Animals.

[31] Yan F., Zhao Q., Li Y., Zheng Z., Kong X., Shu C., Liu Y., Shi Y. (2022). The role of oxidative stress in ovarian aging: A review. J. Ovarian Res..

[32] Miri B., Ghasemi H.A., Hajkhodadadi I., Khaltabadi Farahani A.H. (2022). Effects of low eggshell temperatures during incubation, in ovo feeding of L-arginine, and post-hatch dietary guanidinoacetic acid on hatching traits, performance, and physiological responses of broilers reared at low ambient temperature. Poult. Sci..

[33] Yu L.L., Gao T., Zhao M.M., Lv P.A., Zhang L., Li J.L., Jiang Y., Gao F., Zhou G.H. (2018). In ovo feeding of L-arginine alters energy metabolism in post-hatch broilers. Poult. Sci..

[34] Gao T., Zhao M.M., Li Y.J., Zhang L., Li J.L., Yu L.L., Gao F., Zhou G.H. (2018). Effects of in ovo feeding of L-arginine on the development of digestive organs, intestinal function and post-hatch performance of broiler embryos and hatchlings. J. Anim. Physiol. Anim. Nutr..

[35] Ministry of Agriculture of the People’s Republic of China (2004). NY/T 33-2004 Poultry Feeding Standards.

[36] Rostagno H.S., Albino L.F.T., Donzele J.L., Gomes P.C., de Oliveira R.F., Lopes D.C., Ferreira A.S., de Toledo Barreto S.L., Euclides R.F. (2011). Brazilian Tables for Poultry and Swine.

[37] Sun X., Piao L., Jin H., Nogoy K.M.C., Zhang J., Sun B., Jin Y., Lee D.H., Choi S.H., Smith S.B. (2022). Effects of dietary supplementation of glucose oxidase, catalase, or both on reproductive performance, oxidative stress, fecal microflora and apoptosis in multiparous sows. Anim. Biosci..

[38] Oliva L., Alemany M., Remesar X., Fernández-López J.A. (2019). The Food Energy/Protein Ratio Regulates the Rat Urea Cycle but Not Total Nitrogen Losses. Nutrients.

[39] Khan R.U., Naz S., Ullah H., Ullah Q., Laudadio V., Qudratullah, Bozzo G., Tufarelli V. (2023). Physiological dynamics in broiler chickens under heat stress and possible mitigation strategies. Anim. Biotechnol..

[40] Zahoor I., de Koning D.J., Hocking P.M. (2017). Transcriptional profile of breast muscle in heat stressed layers is similar to that of broiler chickens at control temperature. Genet. Sel. Evol..

[41] Wu X.Y., Wang F.Y., Chen H.X., Dong H.L., Zhao Z.Q., Si L.F. (2023). Chronic heat stress induces lung injury in broiler chickens by disrupting the pulmonary blood-air barrier and activating TLRs/NF-κB signaling pathway. Poult. Sci..

[42] Wang B., Min Z., Yuan J., Zhang B., Guo Y. (2014). Effects of dietary tryptophan and stocking density on the performance, meat quality, and metabolic status of broilers. J. Anim. Sci. Biotechnol..

[43] Wang Q., Liu T., Koci M., Wang Y., Fu Y., Ma M., Ma Q., Zhao L. (2023). Chlorogenic Acid Alleviated AFB1-Induced Hepatotoxicity by Regulating Mitochondrial Function, Activating Nrf2/HO-1, and Inhibiting Noncanonical NF-κB Signaling Pathway. Antioxidants.

[44] Wu S., Wang L., Cui B., Wen X., Jiang Z., Hu S. (2023). Effects of Vitamin A on Growth Performance, Antioxidants, Gut Inflammation, and Microbes in Weaned Piglets. Antioxidants.

[45] Shoveller A.K., Stoll B., Ball R.O., Burrin D.G. (2005). Nutritional and functional importance of intestinal sulfur amino acid metabolism. J. Nutr..

[46] Bin P., Huang R., Zhou X. (2017). Oxidation Resistance of the Sulfur Amino Acids: Methionine and Cysteine. BioMed Res. Int..

[47] Opal S.M., DePalo V.A. (2000). Anti-inflammatory cytokines. Chest.

[48] Yu C., Wang D., Yang Z., Wang T. (2022). Pharmacological Effects of Polyphenol Phytochemicals on the Intestinal Inflammation via Targeting TLR4/NF-κB Signaling Pathway. Int. J. Mol. Sci..

[49] Peng J.L., Bai S.P., Wang J.P., Ding X.M., Zeng Q.F., Zhang K.Y. (2018). Methionine deficiency decreases hepatic lipid exportation and induces liver lipid accumulation in broilers. Poult. Sci..

[50] Willis S., Day C.L., Hinds M.G., Huang D.C. (2003). The Bcl-2-regulated apoptotic pathway. J. Cell Sci..

[51] Spitz A.Z., Gavathiotis E. (2022). Physiological and pharmacological modulation of BAX. Trends Pharmacol. Sci..

[52] Eskandari E., Eaves C.J. (2022). Paradoxical roles of caspase-3 in regulating cell survival, proliferation, and tumorigenesis. J. Cell Biol..

[53] Ohta Y., Kidd M.T., Ishibashi T. (2001). Embryo Growth and Amino Acid Concentration Profiles of Broiler Breeder Eggs, Embryos, and Chicks After In Ovo Administration of Amino Acids. Poult. Sci..

[54] Farias T.M., Cruz F.G.G., Rufino J.P.F., Oliveira Filho P.A.d., Santos A.N.d.A., Bezerra N.d.S., Chaves F.A.d.L., Moda R.F. (2023). Effect of in ovo injection of DL-methionine on hatchability, embryo mortality, hatching weight, blood biochemical parameters and gastrointestinal tract development of breeder chicks. Anim. Biotechnol..

[55] Kop-Bozbay C., Ocak N. (2019). In ovo injection of branched-chain amino acids: Embryonic development, hatchability and hatching quality of turkey poults. J. Anim. Physiol. Anim. Nutr..

[56] Elwan H.A.M., Elnesr S.S., Xu Q., Xie C., Dong X., Zou X. (2019). Effects of In Ovo Methionine-Cysteine Injection on Embryonic Development, Antioxidant Status, IGF-I and TLR4 Gene Expression, and Jejunum Histomorphometry in Newly Hatched Broiler Chicks Exposed to Heat Stress during Incubation. Animals.

